# Supplementary material for: Universal origin of glassy relaxation as recognized by configuration pattern matching
Source: Natl Sci Rev. 2024 Mar 9;11(5):nwae091. doi: 10.1093/nsr/nwae091 (PMC10989661; doi:10.1093/nsr/nwae091)
Supplement: nwae091_Supplemental_File [file nwae091_supplemental_file.pdf]

Supplementary Information for

Universal Origin of Glassy Relaxation as Recognized by Configuration Pattern-matching

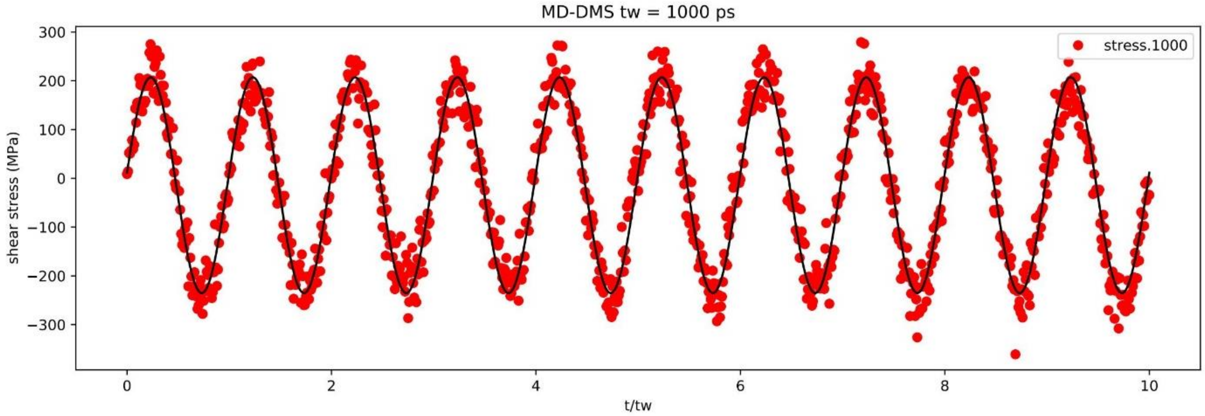

FIG. S1. A typical MD-DMS shear stress response of the $Al_{85}Sm_{15}$ model glass-forming liquids at T = 760 K, p = 0, and with a testing periodic time t_w = 1000 ps. We used 10-cycles for each MD-DMS. The smooth black curve is the fit function via $y = A_0 + A\sin(2\pi t/t_w + \delta)$.

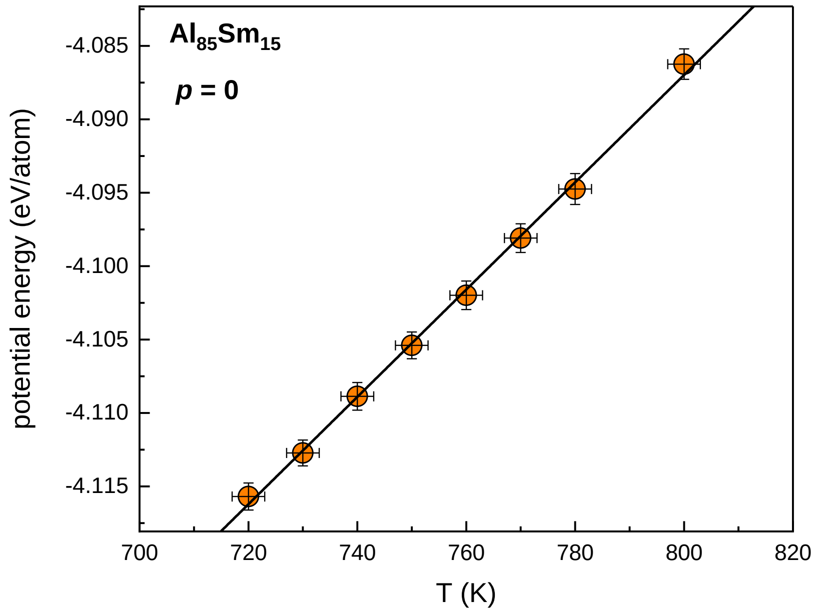

Figure S2. Potential energy as a function of temperature for $Al_{85}Sm_{15}$ model glass-forming liquids. The model liquids at each temperature are equilibrated.

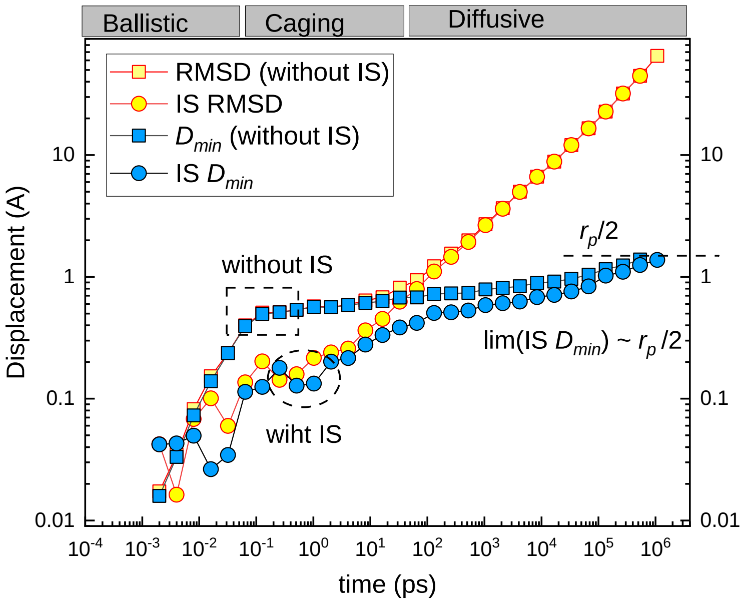

Figure S3. RMSD and IS D_{min} and their "IS-versions" as a function of waiting time for $Al_{85}Sm_{15}$ model glass-forming liquids, at T = 760 K, p = 0, and the liquid is equilibrated for the production run. The dynamics have the ballistic regime, the caging regime, and the diffusion regime as indicated. IS-version refers to that a minimization is first applied to the configuration before the dynamic property calculation.

Interestingly, one finds that the RMSD and IS-RMSD are almost the same at the longer time scale, while they differ substantially in the caging regime. The same trend is also seen in the D_{min} and IS D_{min}.

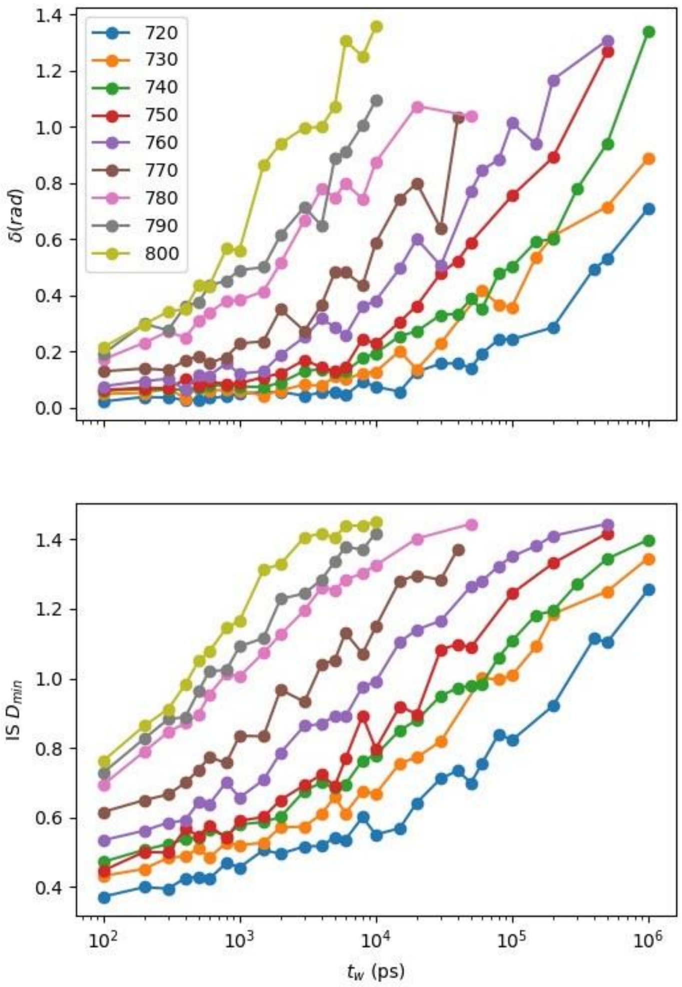

FIG. S4. The phase angle (δ, top panel) and IS D_{min} (bottom panel) for $Al_{85}Sm_{15}$ model glass forming liquids as a function of the t_w at different temperatures as indicated.

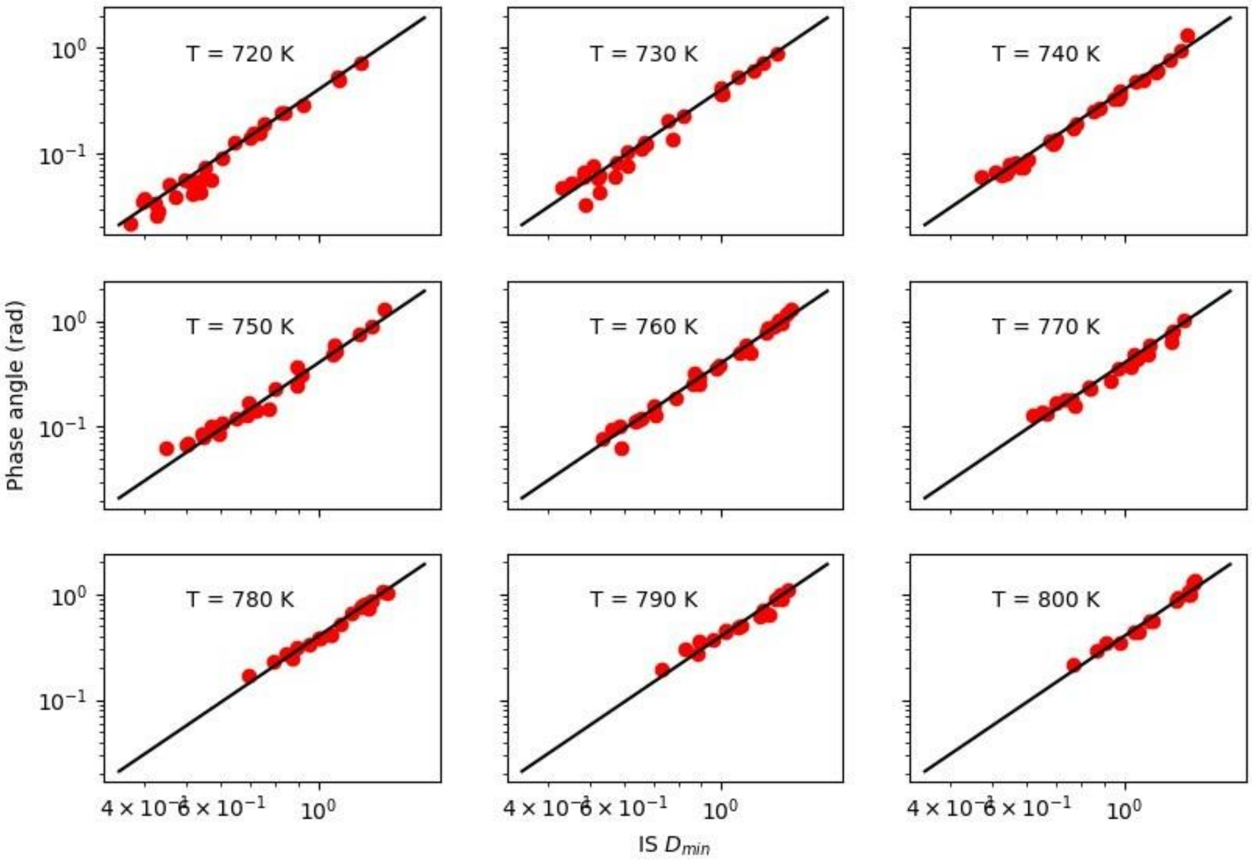

FIG. S5. A facet plot for the relation between IS D_{min} and δ for individual temperature, from 720 to 800K for the Al85Sm15 model glass-forming liquids; the external pressure is $p = 0$.

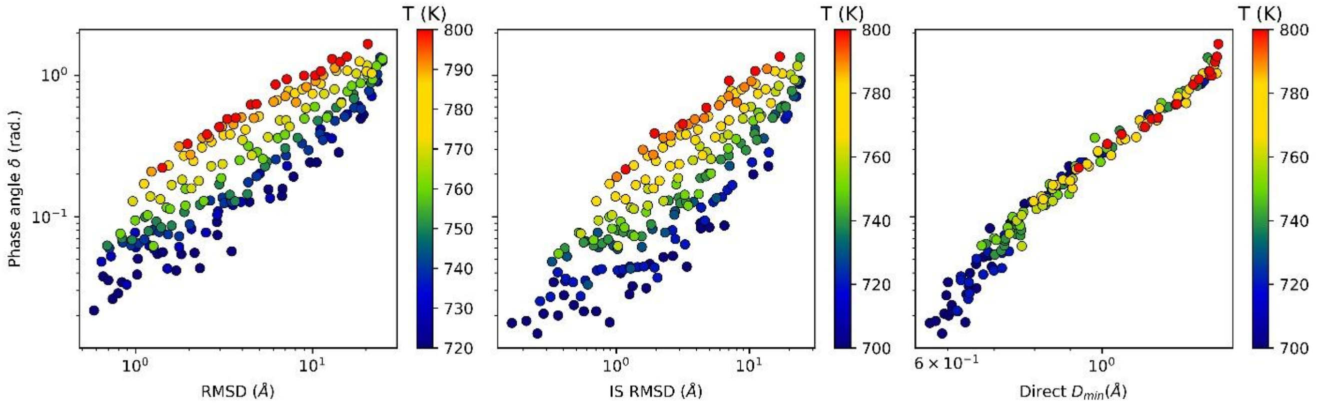

FIG. S6. The plots of the phase angle against RMSD (**left**) and IS RMSD (**middle**) reveal no unified correlation between them respectively. While a correlation between phase angle and direct D_{min} (without evoking IS, **Right panel**) can be seen, the relation is actually not strict power-law.

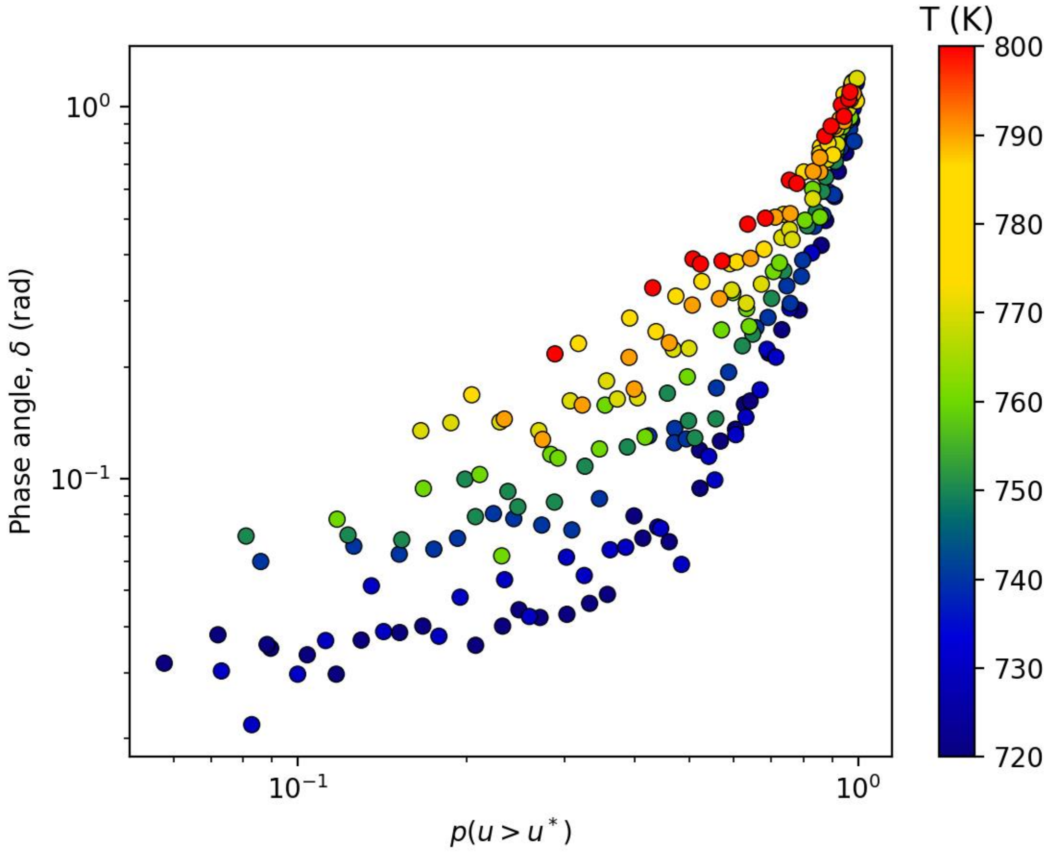

FIG. S7. The plots of the phase angle against the proportion of number of atoms that jump with a distance larger than the half of the mean interatomic distance.

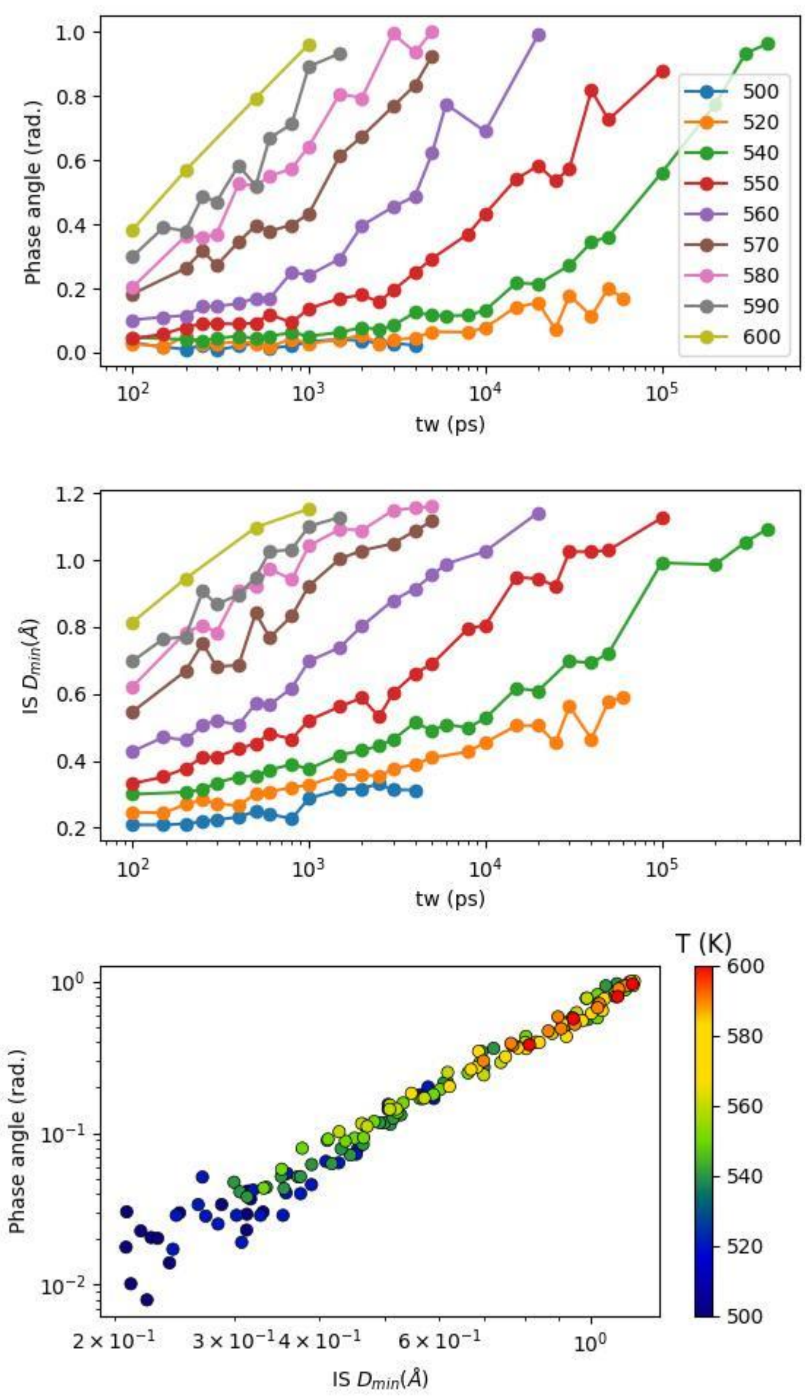

distance

FIG. S8. Relation between IS D_{min} and phase angle in $Ni_{80}P_{20}$. Phase angle (top panel) and IS D_{min} (middle panel) as a function of periodic time t_w for different temperatures as indicated in the top panel; the bottom panel shows the scaling relation between phase angle and IS D_{min}.

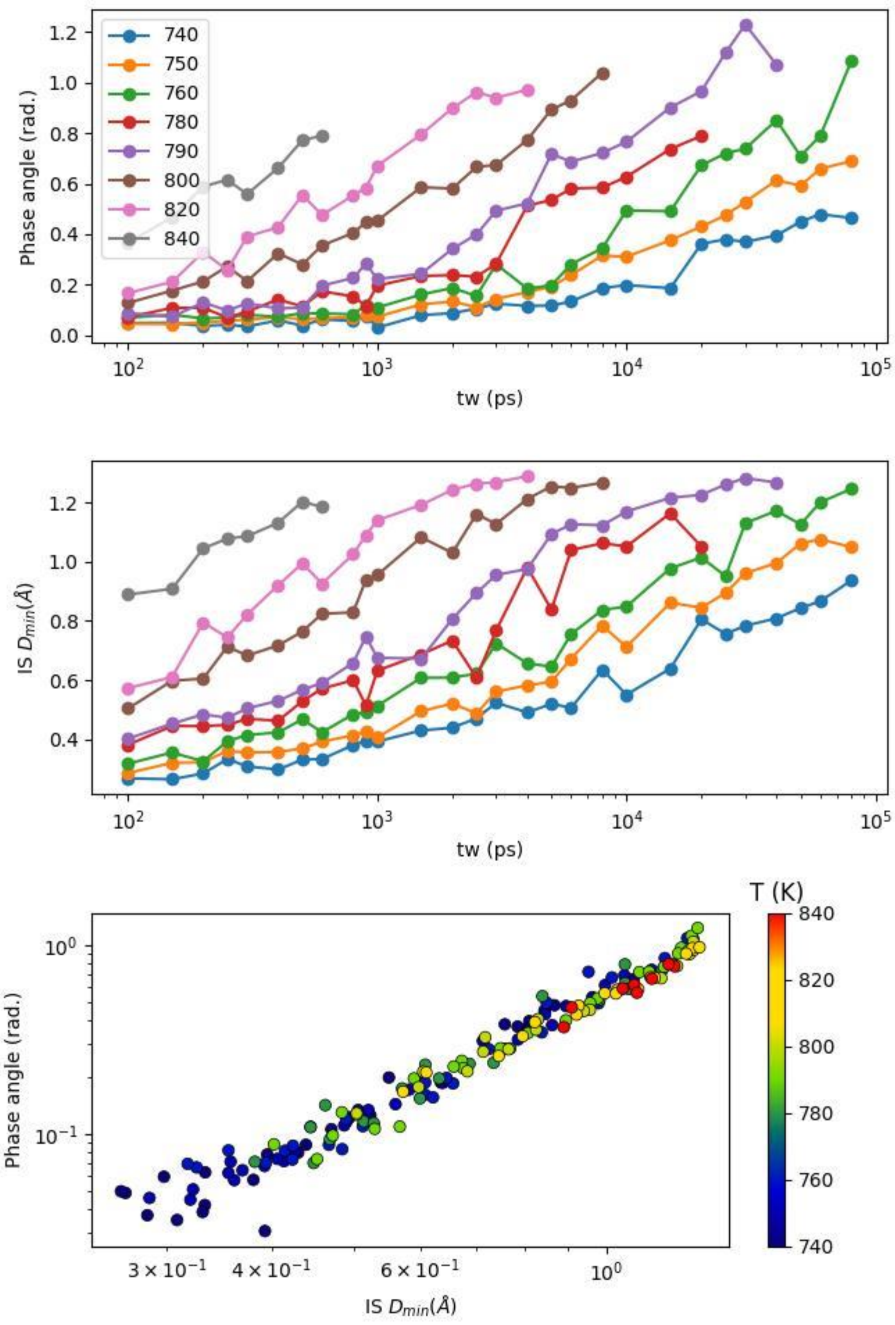

FIG. S9. Relation between IS D_{min} and phase angle in $Pd_{80}Si_{20}$. Phase angle (top panel) and IS D_{min} (middle panel) as a function of periodic time t_w for different temperatures as indicated in the top panel; the bottom panel shows the scaling relation between phase angle and IS D_{min}.

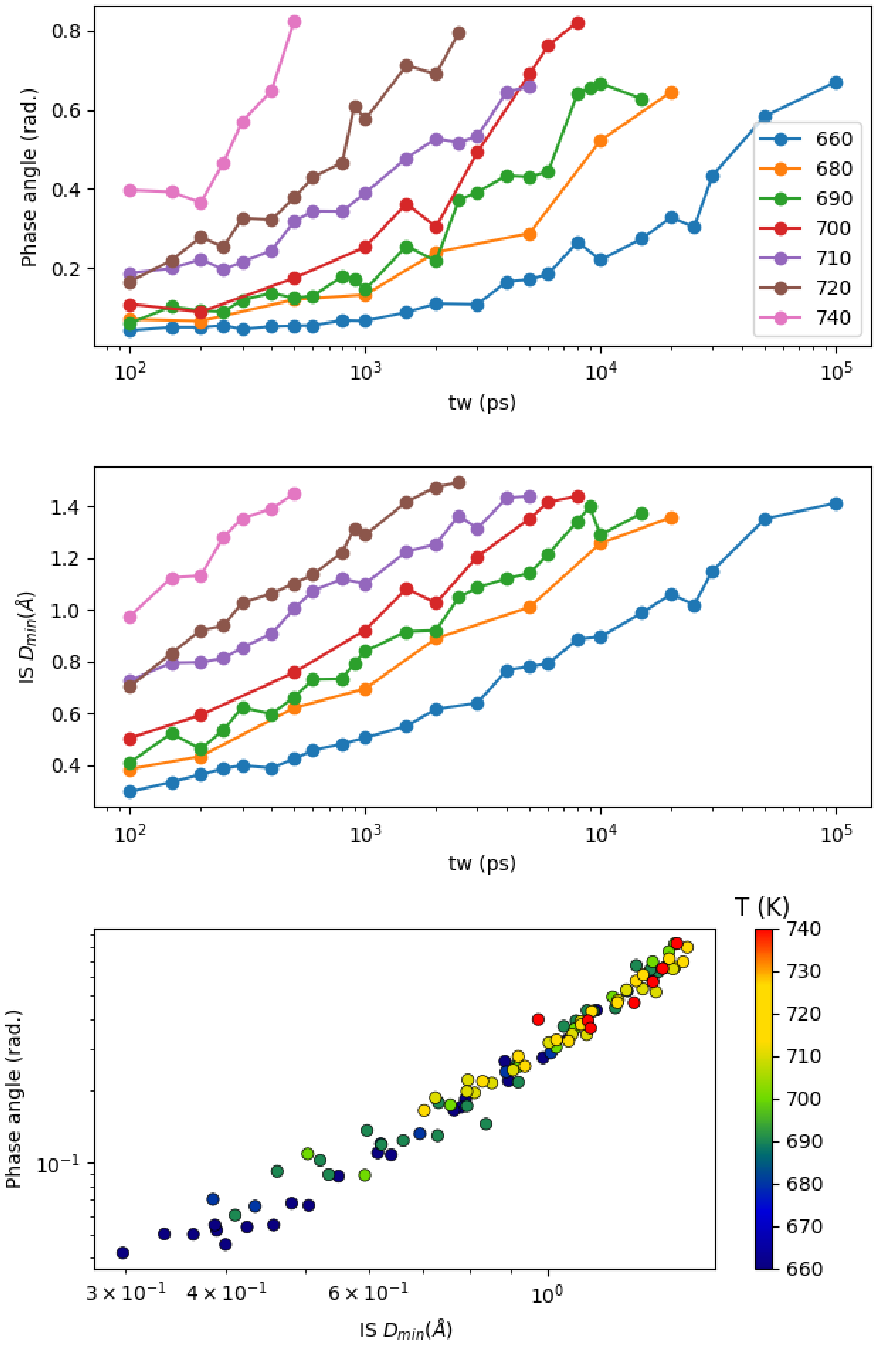

FIG. S10. Relation between IS D_{min} and phase angle in $Y_{65}Cu_{35}$. Phase angle (top panel) and IS D_{min} (middle panel) as a function of periodic time t_w for different temperatures as indicated in the top panel; the bottom panel shows the scaling relation between phase angle and IS D_{min}.

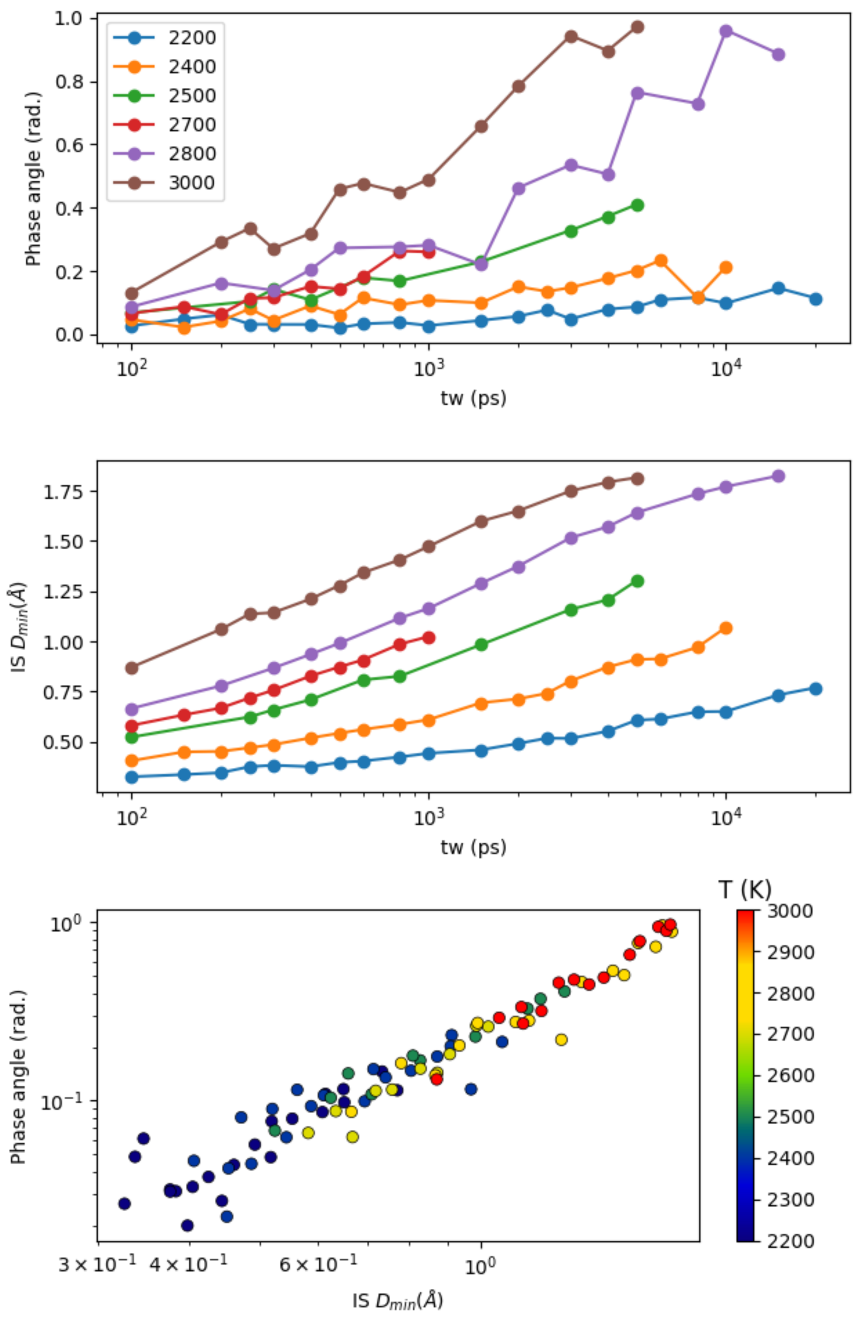

FIG. S11. Relation between IS D_{min} and phase angle in SiO_2. Phase angle (top panel) and IS D_{min} (middle panel) as a function of periodic time t_w for different temperatures as indicated in the top panel; the bottom panel shows the scaling relation between phase angle and IS D_{min}.

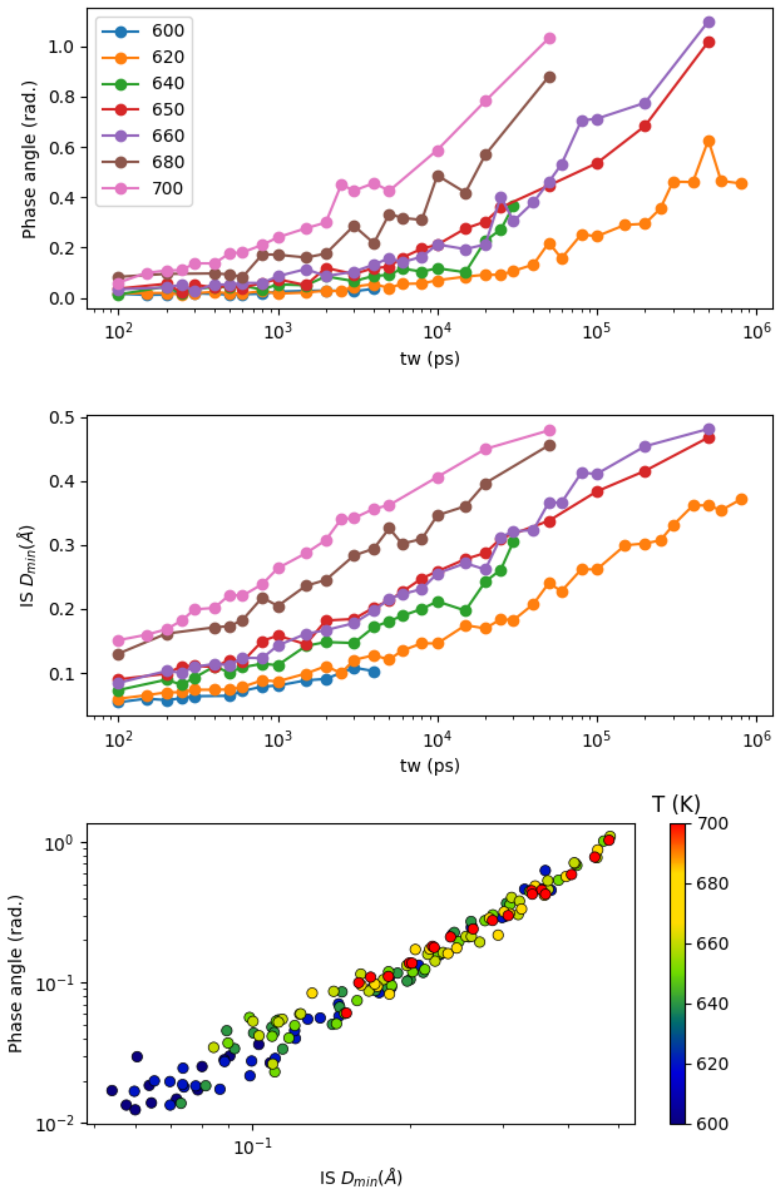

FIG. S12. Relation between IS D_{min} and phase angle in the Kob-Anderson model based on LJ potential. Phase angle (top panel) and IS D_{min} (middle panel) as a function of periodic time t_w for different (1000×T) in the LJ unit as indicated in the top panel; the bottom panel shows the scaling relation between phase angle and IS D_{min}.

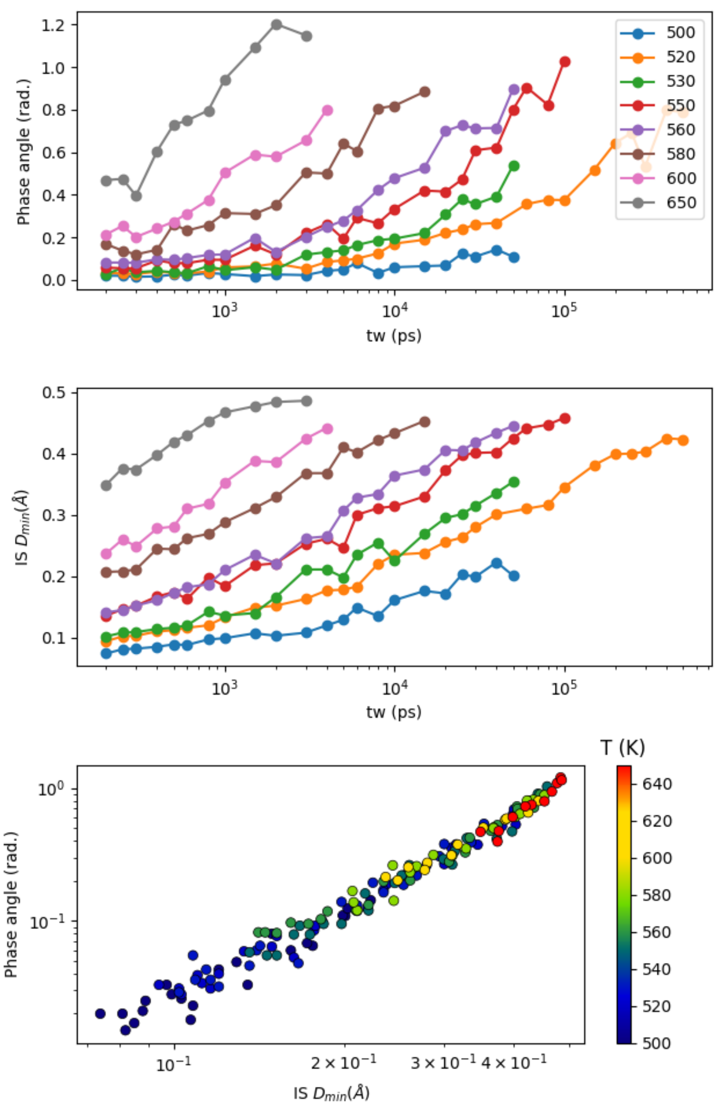

FIG. S13. Relation between IS D_{min} and phase angle in the Weeks-Chandler-Anderson (WCA) model based on LJ potential. Phase angle (top panel) and IS D_{min} (middle panel) as a function of periodic time t_w for different (1000×T) in the LJ unit as indicated in the top panel; the bottom panel shows the scaling relation between phase angle and IS D_{min}.

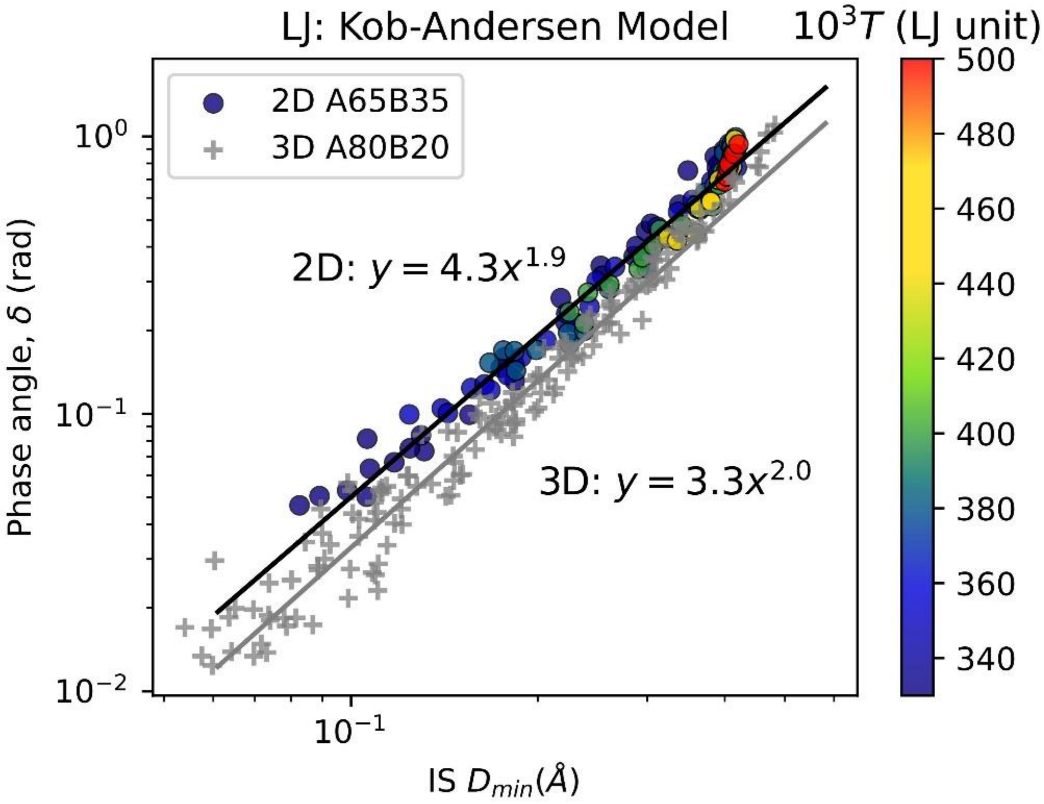

FIG. S14. Comparison between 2D and 3D models for the LJ Kob-Andersen model. It reveals that both 2D and 3D have almost the same power law exponent *b* ~2.

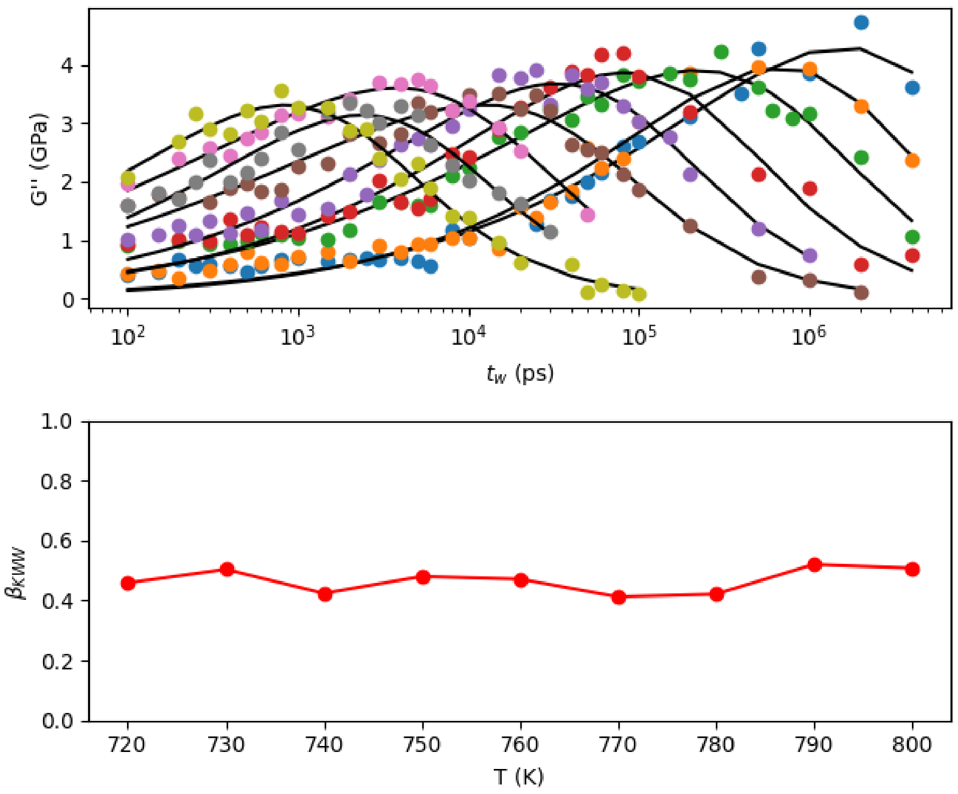

FIG. S15. Fitting the G'' data with KWW equation (top) and the β_{KWW} parameter (bottom) as a function of temperature.

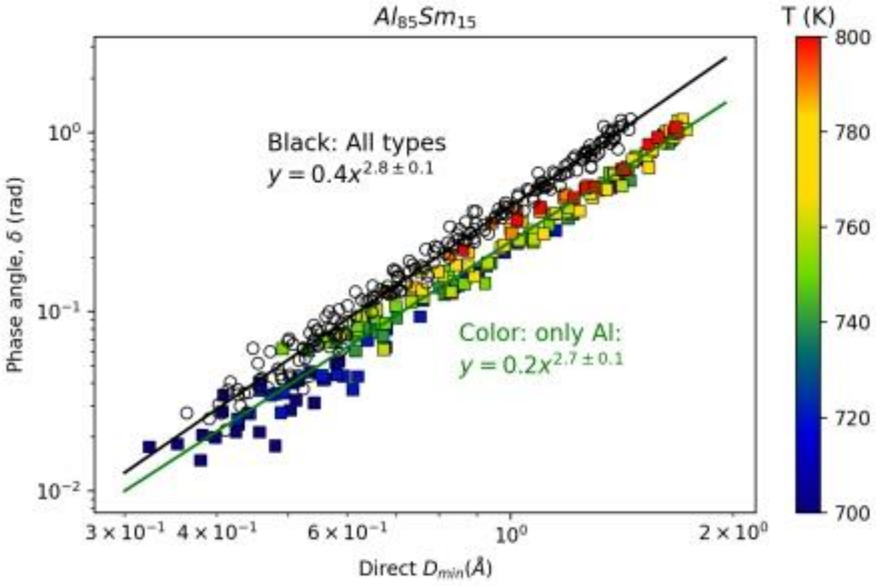

Fig. S16 Relation between phase angle and IS D_{min} for $Al_{85}Sm_{15}$. The color squares are for the Al-atoms only, while the black circles are for all the atoms.

Python script for computation of *IS* D_{min}

It can be accessed via: https://note.youdao.com/s/2RMcW3Oz

```
import numpy as np
import os
import glob
import re
from scipy.optimize import linear_sum_assignment
fn_ref = 'inherent.0'
fn_screen = "screen2.txt"
fp_screep = open(fn_screen, "w")
use_saved_cost_matrix =  not True
save_cost_matrix = not True
save_adjuested_config = True

use_only_selected = not  True
select_type = 1

files = glob.glob("inherent.*")
files.remove(fn_ref)

''''sort by the index
should make sure that this work properly
this is the basis for pair-compare
'''
files.sort(key=lambda l: int(re.findall('\d+', l)[0]))
print(files)

def load_lmp_dump_data(fname):
    lines = open(fname).readlines()
    NAtom = int(lines[3])
    #Boundary length for x,y,z
    x1,x2= list(map(float,lines[5].split())); LX=np.abs(x1-x2)
    y1,y2= list(map(float,lines[6].split())); LY=np.abs(y1-y2)
    z1,z2= list(map(float,lines[7].split())); LZ=np.abs(z1-z2)
    data = np.loadtxt(lines[9:NAtom+9])
    if use_only_selected:
        data = data[np.where(data[:,1]==select_type)]
    return LX, LY, LZ, data

def apply_PBC(M,L):
    M = np.where(M>L/2.0, M-L, M)
    M = np.where(M<-1*L/2.0, M+L, M)
    return M

def vector_to_matrix_function(A,B, L):
    MA= np.array([A for k in range(B.size)])
```

```
    MB= np.array([B for k in range(A.size)]).transpose()
    MC=MA-MB
    MC = apply_PBC(MC, L)
    MC2= MC**2.0
    return MC2

LX0, LY0, LZ0, data0 = load_lmp_dump_data(fn_ref)
x0 = data0[:,2]; y0 = data0[:,3]; z0 = data0[:,4]

result=np.zeros(len(files))

for f_idx, f in enumerate(files):
    fn_calc = f
    label = fn_calc.split(".")[-1]
    f_cost= "cost_matrix.%s" % label
    f_cost_with_ext = f_cost + ".npy"

    LX, LY, LZ, data = load_lmp_dump_data(fn_calc)

    if os.path.isfile(f_cost_with_ext) and use_saved_cost_matrix:
        cost = np.load(f_cost_with_ext)
    else:
        x= data[:,2]; y =data[:,3]; z = data[:,4]
        MX= vector_to_matrix_function(x0,x,LX)
        MY= vector_to_matrix_function(y0,y,LY)
        MZ= vector_to_matrix_function(z0,z,LZ)
        M_dis2 = MX+MY+MZ
        if save_cost_matrix:  np.save(f_cost,M_dis2)
    cost=M_dis2
    row_ind, col_ind = linear_sum_assignment(cost)
    new_data= data[col_ind]
    N = len(data)
    new_data[:,0] = np.arange(1, N+1)
    if save_adjuested_config:
        headstring=open(fn_calc).readlines()[:9]
        fp_res=open('adjusted.%s'%label,'w')
        headstring[3]='%s\n' %len(new_data)
        fp_res.writelines(headstring)
        np.savetxt(fp_res, new_data,fmt="%d %d %lf %lf %lf")
        fp_res.close()
    s = cost[row_ind, col_ind].mean()
    s_perAtom = np.sqrt(s)
    print("%s %lf" % (label, s_perAtom))
    fp_screep.write("%s %lf\n" % (label, s_perAtom))
    fp_screep.flush()
    '''''
    The following line is used for pair compare
    e.g., 0-1,1-2,..
```

```
    '''
    x0 = x; y0 = y; z0 = z
    result[f_idx] = s_perAtom
fp_screep.close()
print('Result: Meanvalue = %.4f  Std =%.4f, Nsamp = %d' %(result.mean(),
  result.std(), len(result)) )
```
